# Optimization of initial dosage of quetiapine in schizophrenic patients: effects of fluvoxamine or duloxetine coadministration

**DOI:** 10.3389/fphar.2024.1496043

**Published:** 2024-11-20

**Authors:** Xiao Chen, Yue Zhang, Di Yin, Ying-Wei Jin, Su-Mei He, Chen-Xu Liu, Cun Zhang, Dong-Dong Wang

**Affiliations:** ^1^ School of Nursing, Xuzhou Medical University, Xuzhou, Jiangsu, China; ^2^ Jiangsu Key Laboratory of New Drug Research and Clinical Pharmacy and School of Pharmacy, Xuzhou Medical University, Xuzhou, Jiangsu, China; ^3^ Department of Pharmacy, Wuxi Maternity and Child Health Care Hospital, Wuxi, Jiangsu, China; ^4^ Department of Pharmacy, The Suqian Clinical College of Xuzhou Medical University, Suqian, Jiangsu, China; ^5^ Department of Pharmacy, Suzhou Research Center of Medical School, Suzhou Hospital, Affiliated Hospital of Medical School, Nanjing University, Suzhou, Jiangsu, China; ^6^ Department of Pharmacy, Shenzhen Hospital, Southern Medical University, Shenzhen, China; ^7^ Department of Pharmacy, Xuzhou Oriental Hospital Affiliated to Xuzhou Medical University, Xuzhou, Jiangsu, China

**Keywords:** optimal initial dosage, quetiapine, schizophrenic patient, fluvoxamine, duloxetine, drug–drug interactions

## Abstract

**Objective:**

Although quetiapine has been approved for use in schizophrenic patients, its individualized dosage regimen remains unclear, especially with respect to drug–drug interactions (DDIs). Thus, we investigated the potential DDIs and optimal initial dosage of quetiapine in schizophrenic patients based on population pharmacokinetics (PPK).

**Methods:**

Ninety-six schizophrenic patients treated with quetiapine were included to establish the PPK model, which also includes coadministration of multiple drugs.

**Results:**

It was found that the patient weights and fluvoxamine or duloxetine coadministration affected quetiapine clearance in schizophrenic patients. Without fluvoxamine or duloxetine coadministration, 16 and 12 mg/kg/day of quetiapine were recommended to schizophrenic patients whose weights were in the ranges of 40–50 and 50–120 kg, respectively. With fluvoxamine coadministration, 8 mg/kg/day of quetiapine was recommended to patients with weights in the range of 40–120 kg. With duloxetine coadministration, 8 mg/kg/day of quetiapine was recommended to patients with weights in the 40–120 kg range. With simultaneous coadministration of fluvoxamine and duloxetine, 4 mg/kg/day of quetiapine was recommended to patients with weights in the 40–120 kg range.

**Conclusion:**

The present study was a pilot effort at investigating the potential DDIs and optimal initial dosage of quetiapine in schizophrenic patients based on PPK. The initial dosages of quetiapine administered to the patients were optimized according to the coadministration of fluvoxamine or duloxetine.

## 1 Introduction

Schizophrenia is a mental disease occurring in late adolescence and young adulthood; it is often accompanied by sensory, thinking, emotional, will-based, and behavioral disorders in combination with social or occupational defects and is considered to be one of the most serious mental diseases (Charlson et al., 2018; Jauhar et al., 2022). The clinical treatment of schizophrenia involves severe challenges because of its complex etiology, interlaced symptoms, and high recurrence rate, for which drug therapy remains the main mode of treatment at present (Li et al., 2023; Wang et al., 2024; Zhang et al., 2024a; Zhang et al., 2024b).

Quetiapine is a dibenzothiazepine derivative containing low-affinity dopamine D_2_ and serotonin 5-HT_2A_ antagonist belonging to atypical antipsychotics (Cheer and Wagstaff, 2004; Hao et al., 2023); it has been approved for use in schizophrenia and is presently the most commonly prescribed antipsychotic medication among adults aged 20–64 years in almost 71% of the countries globally (Kasper and Muller-Spahn, 2000; Cheer and Wagstaff, 2004; Hojlund et al., 2021; Hao et al., 2023).

In terms of pharmacokinetics, quetiapine is mainly metabolized by CYP3A4, CYP2C19, and CYP2D6 (Bakken et al., 2012; Cabaleiro et al., 2015; Xu et al., 2016; Liu et al., 2021; Stauble et al., 2021; Rohail et al., 2023; Yau et al., 2023). When drug combinations are used clinically, especially when there is inhibition or induction of CYP3A4, CYP2C19, or CYP2D6, quetiapine could have significant variations in terms of clearance and drug concentration. Quetiapine has been reported to have many interactions, especially with drugs used against cardiovascular diseases (Siwek et al., 2020) and other drugs such as erythromycin (Li et al., 2005), clarithromycin (Schulz-Du Bois et al., 2008), aprepitant (Patel et al., 2017), lovastatin (Furst et al., 2002), as well as medicinal products and diet supplements containing herbal extracts or grapefruit (Cinderella et al., 2021). From a clinical perspective, low concentrations of quetiapine have been associated with reduced drug effects and poor psychiatric control, whereas high quetiapine concentrations may cause adverse reactions (Hao et al., 2023). Thus, the present study was aimed at investigating the potential drug–drug interactions (DDIs) and optimal initial dosage of quetiapine in schizophrenic patients based on population pharmacokinetics (PPK).

## 2 Methods

### 2.1 Information collection

Schizophrenic patients treated with quetiapine at the Xuzhou Oriental Hospital Affiliated to Xuzhou Medical University between July 2020 and November 2023 were enrolled in this investigation, which was a single-center study. We assessed quetiapine concentrations for therapeutic drug monitoring (TDM) while also collecting the physiological and biochemical indexes of the patients as well as information regarding drug combinations. The present study was approved by the Research Ethics Committee of Xuzhou Oriental Hospital Affiliated to Xuzhou Medical University.

### 2.2 Modeling

We constructed a PPK model using the non-linear mixed-effect modeling (NONMEM) approach using the apparent oral clearance (CL/F), apparent volume of distribution (V/F), and absorption rate constant (Ka) fixed at 1.46/h (Zhou et al., 2015) as the assessment parameters.

Equation 1 is the expression for the interindividual variability:

$$B_{i}=\text{TV}\left(B\right)\times \exp\left(\eta_{i}\right),$$ (1)

where B_i_ is the individual parameter, TV(B) is the typical individual parameter, and η_i_ indicates symmetrical distribution.

Equation 2 gives the expression for the random residual variability:

$$D_{i}=F_{i}+F_{i*}\varepsilon_{1}+\varepsilon_{2},$$ (2)

where D_i_ is the observed concentration, F_i_ is the individual predicted concentration, and ε_n_ indicates symmetrical distribution.

Equation 3 shows the relationship of the pharmacokinetic parameters with weight:

$$H_{i}=H_{\text{std}}\times {\left(L_{i}/L_{\text{std}}\right)}^{N},$$ (3)

where H_i_ is the *i*th individual parameter, L_i_ is the *i*th individual weight, L_std_ is the standard weight of 70 kg, and H_std_ is the typical individual parameter. The variable N is the allometric coefficient, which is 0.75 for CL/F and 1 for V/F (Anderson and Holford, 2008).

Equations (4, 5) show the pharmacokinetic parameters for the continuous and categorical covariates, respectively:

$$O_{i}=\text{TV}\left(O\right)\times {\left(Z_{i}/Z_{m}\right)}^{p},$$ (4)

$$O_{i}=\text{TV}\left(O\right)\times \left(1+p\times Z_{i}\right),$$ (5)

where O_i_ is the individual parameter, TV(O) is the typical individual parameter, p is the parameter to be estimated, Z_i_ is the covariate of the *i*th individual, and Z_m_ is the population median for the covariate.

A stepwise method was used to analyze the covariates in the PPK model of quetiapine in schizophrenic patients. In this process, a decrease in the objective function value (OFV) by more than 3.84 (*P*< 0.05) was accepted as the inclusion standard and an increase in OFV by more than 6.63 (*P*< 0.01) was considered as the exclusion standard.

### 2.3 Model evaluation

The final model was evaluated through visualization, and the bootstrap method was used to compare the final model parameters.

### 2.4 Simulation

Monte Carlo simulations were conducted regarding the optimal quetiapine concentrations for schizophrenic patients given that the recommended therapeutic window for quetiapine was 100–500 ng/mL (Lin et al., 2024). It was found that the patient weight as well as fluvoxamine or duloxetine coadministration significantly impacted quetiapine clearance in the patients. Hence, based on the coadministration of fluvoxamine or duloxetine, four different conditions were simulated in the present study: schizophrenic patients without fluvoxamine or duloxetine coadministration, schizophrenia patients with fluvoxamine coadministration, schizophrenic patients with duloxetine coadministration, and schizophrenic patients administered both fluvoxamine and duloxetine. Each condition was simulated with 1,000 virtual schizophrenic patients under five weight groups (40, 60, 80, 100, and 120 kg) and eight dosage groups (1, 4, 8, 12, 16, 20, 24, and 28 mg/kg/day) each. The probability of achieving the target concentration was selected as the evaluation criterion, and the probability of exceeding the upper limit of the treatment window (500 ng/mL) over 1,000 simulated concentrations was deemed the safety evaluation measure.

## 3 Results

### 3.1 Patient information

Ninety-six schizophrenic patients treated with quetiapine (immediate-release tablets) and 154 quetiapine concentrations were included in this study to establish the PPK model; the patients included 52 men and 44 women of age 43.53 ± 14.17 years weighing 70.88 ± 16.84 kg who were coadministered multiple drugs. The demographic data and drug combinations of the patients given quetiapine are summarized in Tables 1, 2, respectively.

**TABLE 1:** Demographic data on the schizophrenic patients treated with quetiapine (n = 96).

Characteristic	Mean ± SD
Gender (men/women)	52/44
Age (years)	43.53 ± 14.17
Weight (kg)	70.88 ± 16.84
Albumin (g/L)	41.39 ± 3.27
Globulin (g/L)	27.14 ± 3.44
Alanine transaminase (IU/L)	29.57 ± 24.97
Aspartate transaminase (IU/L)	22.02 ± 11.78
Creatinine (μmol/L)	63.64 ± 15.19
Urea (mmol/L)	4.52 ± 1.30
Total protein (g/L)	68.53 ± 4.86
Total cholesterol (mmol/L)	4.55 ± 1.08
Triglyceride (mmol/L)	2.08 ± 1.30
Direct bilirubin (μmol/L)	2.62 ± 1.44
Total bilirubin (μmol/L)	8.07 ± 3.36
Hematocrit (%)	39.22 ± 4.81
Hemoglobin (g/L)	129.01 ± 17.10
Mean corpuscular hemoglobin (pg)	29.56 ± 2.37
Mean corpuscular hemoglobin concentration (g/L)	328.64 ± 10.87

**TABLE 2:** Drug combinations administered to the schizophrenic patients (n = 96).

Drug	Category	N	Drug	Category	N
Acarbose capsules	0	91	Lorazepam tablets	0	80
	1	5		1	16
Agomelatine tables	0	94	Metformin hydrochloride tablets	0	80
	1	2		1	16
Alprazolam tablets	0	87	Nifedipine sustained-release tablets	0	93
	1	9		1	3
Amlodipine besylate tablets	0	94	Oxazepam tablets	0	90
	1	2		1	6
Aripiprazole tablets	0	80	Perphenazine tablets	0	92
	1	16		1	4
Aspirin enteric-coated tablets	0	92	Propranolol hydrochloride tablets	0	79
	1	4		1	17
Atorvastatin calcium tablets	0	91	Risperidone tablets	0	78
	1	5		1	18
Clonazepam tablets	0	89	Silymarin capsules	0	94
	1	7		1	2
Clozapine tablets	0	77	Sodium valproate sustained-release tablets	0	74
	1	19		1	22
Duloxetine hydrochloride enteric-coated capsules	0	94	Spironolactone tablets	0	93
	1	2		1	3
Fluvoxamine maleate tablets	0	94	Trihexyphenidyl hydrochloride tablets	0	73
	1	2		1	23
Glimepiride tablets	0	91	Valsartan capsules	0	93
	1	5		1	3
Lithium carbonate sustained-release tablets	0	80	Zopiclone tablets	0	87
	1	16		1	9

Category, 0: without drug, 1: with drug; N, number of patients.

### 3.2 Modeling

The patient weight as well as coadministration of fluvoxamine or duloxetine affected quetiapine clearance in the schizophrenic patients. At the same weight, the quetiapine clearance rates were 1, 0.464, 0.463, and 0.214832 in the patients without fluvoxamine or duloxetine coadministration, with fluvoxamine coadministration, with duloxetine coadministration, and with both fluvoxamine and duloxetine coadministration, respectively. Thus, the PPK model of quetiapine in the schizophrenic patients is as follows (Equations 6, 7)

$$\text{CL}/F=118\times {\left(\text{weight}/70\right)}^{0.75}\times \left(1-0.536\times \text{FLU}\right)\times \left(1-0.537\times \text{DUL}\right)$$ (6)

$$V/F=2460\times \left(\text{weight }/70\right),$$ (7)

where CL/F is the apparent oral clearance, and V/F is the apparent volume of distribution; FLU and DUL refer to fluvoxamine and duloxetine, respectively. When the schizophrenic patients were administered fluvoxamine or duloxetine, the values of FLU and DUL were 1; otherwise, FLU and DUL were set to 0.

### 3.3 Evaluation

**FIGURE 1:**
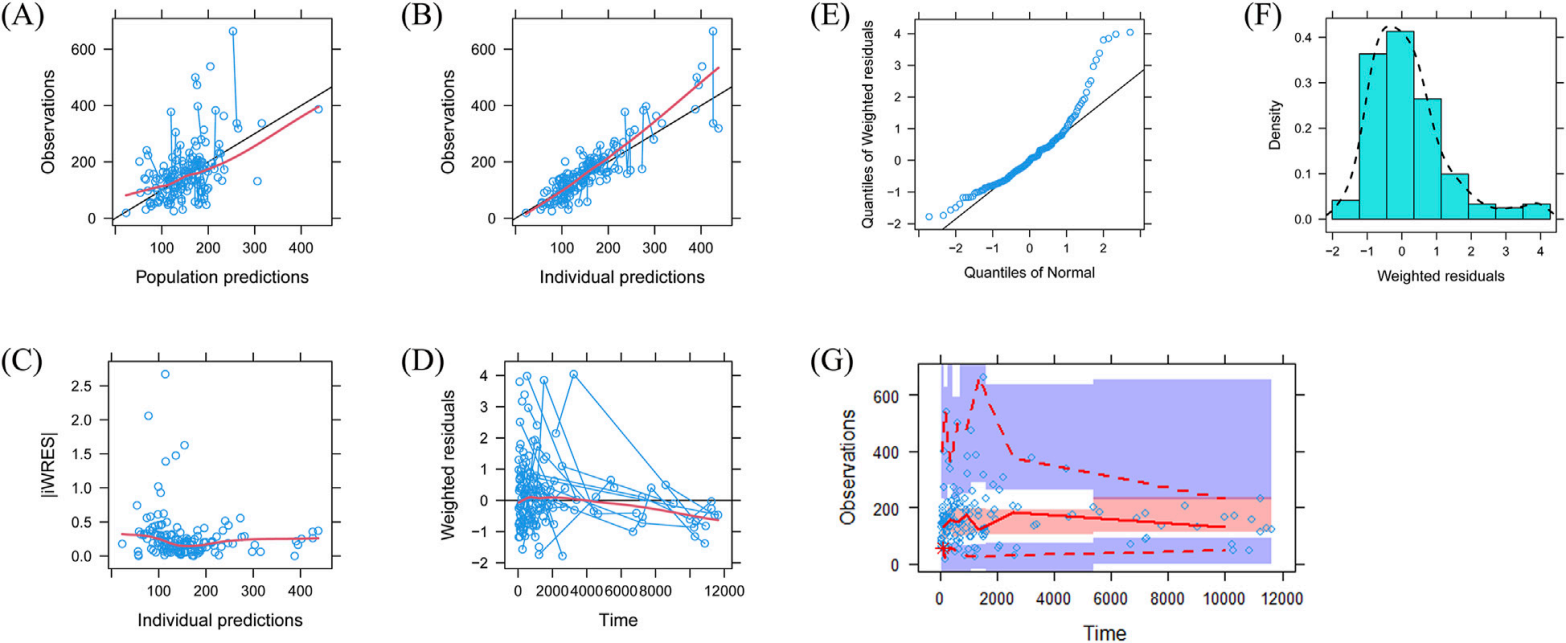
Model evaluations: **(A)** observations vs. population predictions; **(B)** observations vs. individual predictions; **(C)** absolute value of the weighted residuals of the individuals (│iWRES│) vs. individual predictions; **(D)** weighted residuals vs. time; **(E)** quantiles of weighted residuals vs. normal quantiles; **(F)** density vs. weighted residuals; **(G)** visual predictive check of the model.

**FIGURE 2:**
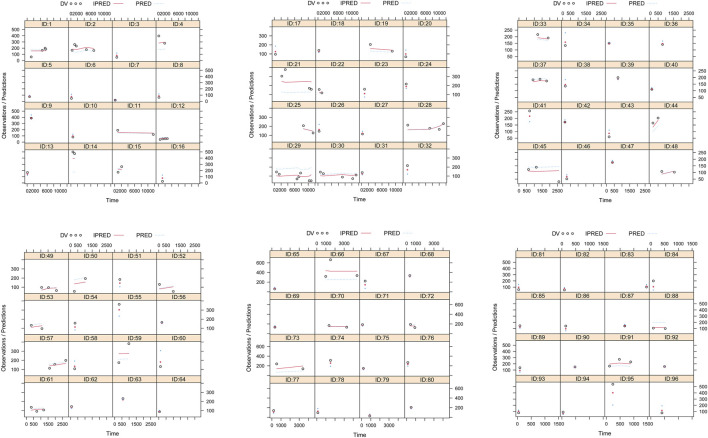
Plots of the individual subjects. ID, patient ID number; DV, measured concentration; IPRED, individual predicted value; PRED, population predicted value.

The quetiapine PPK model observations are shown in Figure 1A–G, which indicate that the quetiapine concentrations are well predicted. Figure 2 shows the plots of the individuals and shows that the quetiapine PPK model accurately predicts the quetiapine concentrations at the individual level. The bootstrap validation results are shown in Table 3, which indicates that the final model is accurate and reliable.

**TABLE 3:** Parameter estimates of the quetiapine final model and bootstrap validations in schizophrenic patients.

			Bootstrap		
Parameter	Estimate	SE (%)	Median	90% Confidence interval	Bias (%)
CL/F (L/h)	118	6.9	117	[100, 130]	−0.85
V/F (L)	2,460	33.5	2,505	[1,222, 5,163]	1.83
Ka (h^-1^)	1.46 (fixed)	—	—	—	—
θ_FLU_	−0.536	4.7	−0.535	[–0.579, −0.487]	−0.19
θ_DUL_	−0.537	12.0	−0.533	[–0.642, −0.417]	−0.74
ω_CL/F_	0.333	12.9	0.325	[0.230, 0.410]	−2.40
σ_1_	0.267	15.3	0.258	[0.168, 0.327]	−3.37
σ_2_	29.917	34.7	31.780	[2.380, 49.785]	6.23

The 90% confidential interval is displayed as the 5th to 95th percentile of the bootstrap estimates. CL/F, apparent oral clearance (L/h); V/F, apparent volume of distribution (L); Ka, absorption rate constant (h^-1^); θ_FLU_ and θ_DUL_ are the coefficients of fluvoxamine and duloxetine, respectively; ω_CL/F_, inter-individual variability of CL/F; σ_1_, residual variability with proportional error; σ_2_, residual variability with additive error; Bias, prediction error given as [(median–estimate) × 100% / estimate].

### 3.4 Recommended dosage

**FIGURE 3:**
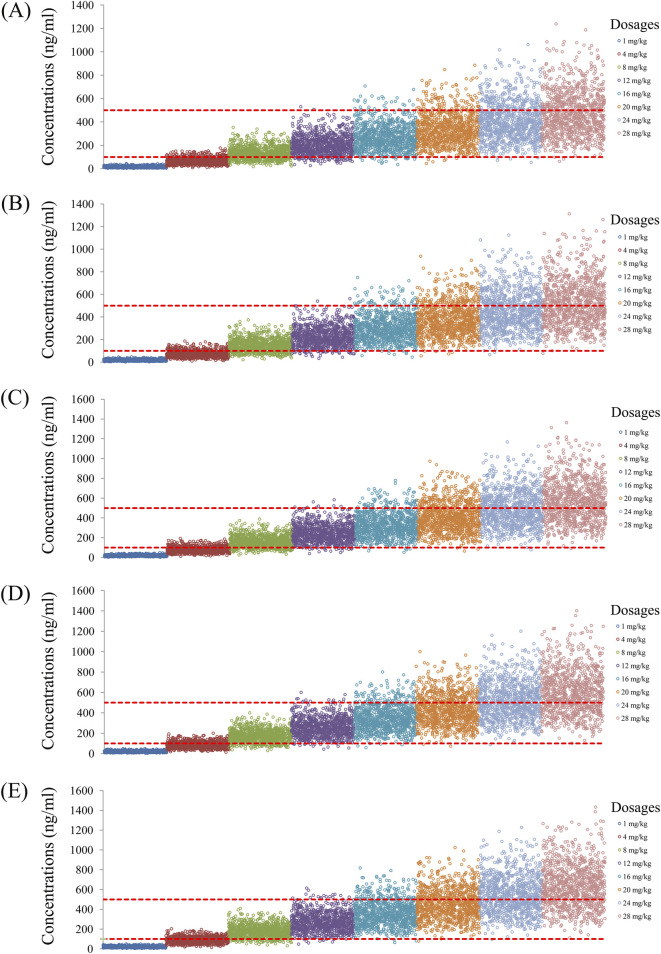
Simulated quetiapine concentrations without fluvoxamine or duloxetine coadministration for schizophrenic patients of weights **(A)** 40 kg, **(B)** 60 kg, **(C)** 80 kg, **(D)** 100 kg, and **(E)** 120 kg.

**FIGURE 4:**
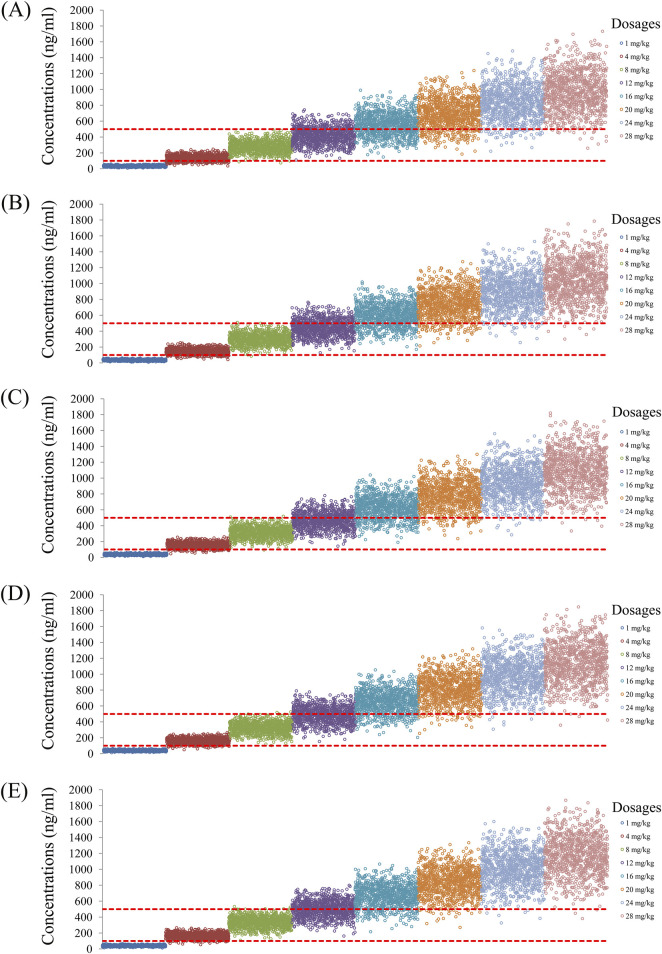
Simulated quetiapine concentrations with fluvoxamine coadministration for schizophrenic patients of weights **(A)** 40 kg, **(B)** 60 kg, **(C)** 80 kg, **(D)** 100 kg, and **(E)** 120 kg.

**FIGURE 5:**
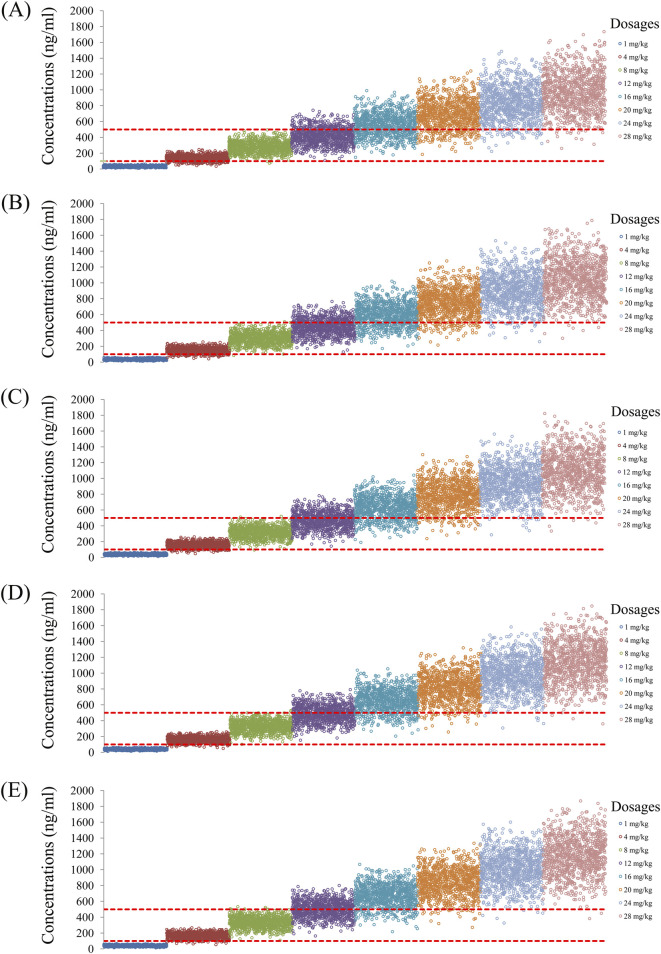
Simulated quetiapine concentrations with duloxetine coadministration for schizophrenic patients of weights **(A)** 40 kg, **(B)** 60 kg, **(C)** 80 kg, **(D)** 100 kg, and **(E)** 120 kg.

**FIGURE 6:**
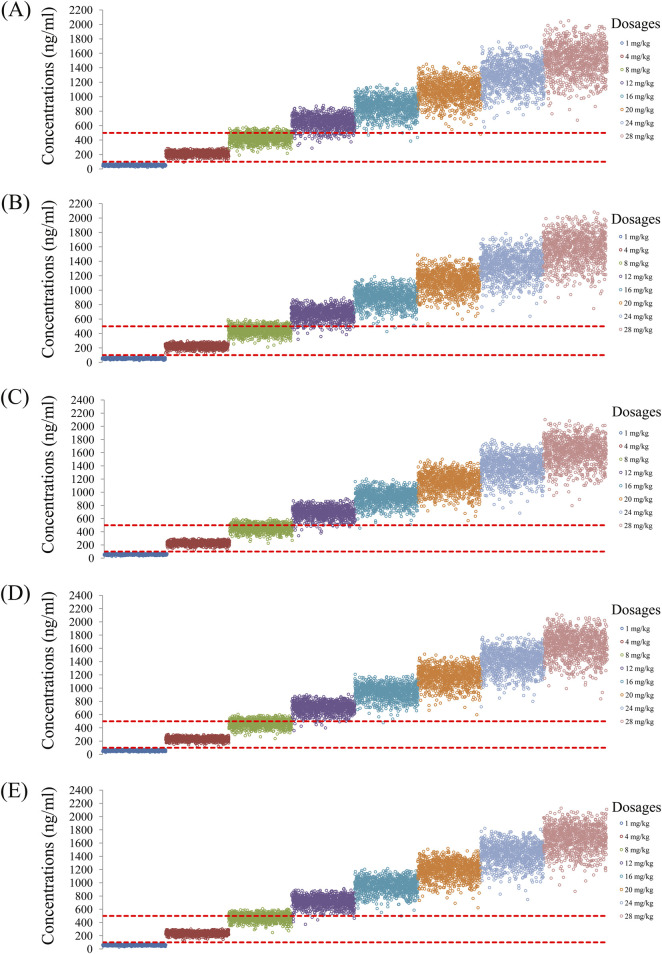
Simulated quetiapine concentrations with coadministration of both fluvoxamine and duloxetine for schizophrenic patients of weights **(A)** 40 kg, **(B)** 60 kg, **(C)** 80 kg, **(D)** 100 kg, and **(E)** 120 kg.

**FIGURE 7:**
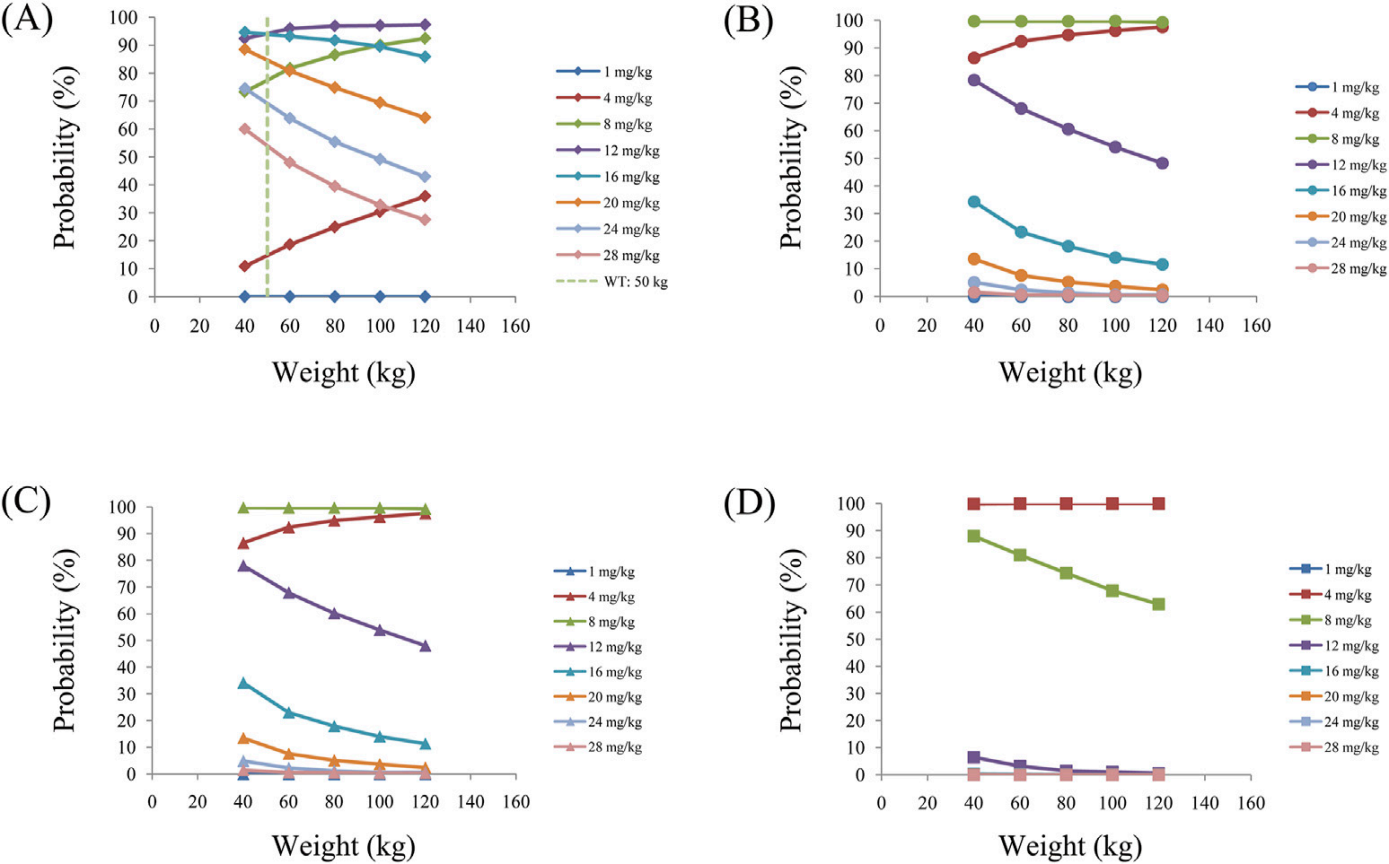
Probabilities of achieving the target concentrations of quetiapine in schizophrenic patients **(A)** without fluvoxamine or duloxetine coadministration, **(B)** with fluvoxamine coadministration, **(C)** with duloxetine coadministration, and **(D)** with coadministration of both fluvoxamine and duloxetine.

As noted earlier, four different conditions were simulated in this study, namely schizophrenia patients without fluvoxamine or duloxetine coadministration, with fluvoxamine coadministration, with duloxetine coadministration, and with both fluvoxamine and duloxetine coadministration, whose results are shown in Figures 3–6, respectively. The probabilities of achieving the target concentrations of quetiapine in the schizophrenic patients under the four conditions are demonstrated in Figure 7; here Figures 7A–D are the results for the schizophrenic patients without fluvoxamine or duloxetine coadministration, with fluvoxamine coadministration, with duloxetine coadministration, and with both fluvoxamine and duloxetine coadministration, respectively. The optimal initial dosages of quetiapine in the schizophrenic patients are summarized in Table 4. Accordingly, without fluvoxamine or duloxetine coadministration, 16 and 12 mg/kg/day of quetiapine are recommended to patients whose weights are in the 40–50 and 50–120 kg ranges, for which the probabilities of achieving the target concentrations are 94.0%–94.7% and 94.0%–97.3%, respectively. For fluvoxamine coadministration, 8 mg/kg/day of quetiapine is recommended to patients in the weight range of 40–120 kg, for which the probability of achieving the target concentration is 99.3%–99.8%. For duloxetine coadministration, 8 mg/kg/day of quetiapine is recommended to patients with weights in the range of 40–120 kg, for which the probability of achieving the target concentration is 99.3%–99.8%. For both fluvoxamine and duloxetine coadministration, 4 mg/kg/day of quetiapine is recommended to patients with weights in the range of 40–120 kg, for which the probability of achieving the target concentration is 99.9%–100.0%.

**TABLE 4:** Initial dosage recommendations of quetiapine for schizophrenic patients.

Without fluvoxamine				With fluvoxamine			
Without duloxetine				Without duloxetine			
Body weight (kg)	Dosage (mg/kg/day)	Probability to achieve the target concentrations (%)	Probability to exceed the upper limit of the target concentrations (%)	Body weight (kg)	Dosage (mg/kg/day)	Probability to achieve the target concentrations (%)	Probability to exceed the upper limit of the target concentrations (%)
[40–50)	16	94.0–94.7	3.1–4.6	[40–120]	8	99.3–99.8	0–0.7
[50–120]	12	94.0–97.3	0.2–1.8				

With duloxetine				With duloxetine			
Body weight (kg)	Dosage (mg/kg/day)	Probability to achieve the target concentrations (%)	Probability to exceed the upper limit of the target concentrations (%)	Body weight (kg)	Dosage (mg/kg/day)	Probability to achieve the target concentrations (%)	Probability to exceed the upper limit of the target concentrations (%)
[40–120]	8	99.3–99.8	0–0.7	[40–120]	4	99.9–100.0	0

### 3.5 Safety evaluation

**FIGURE 8:**
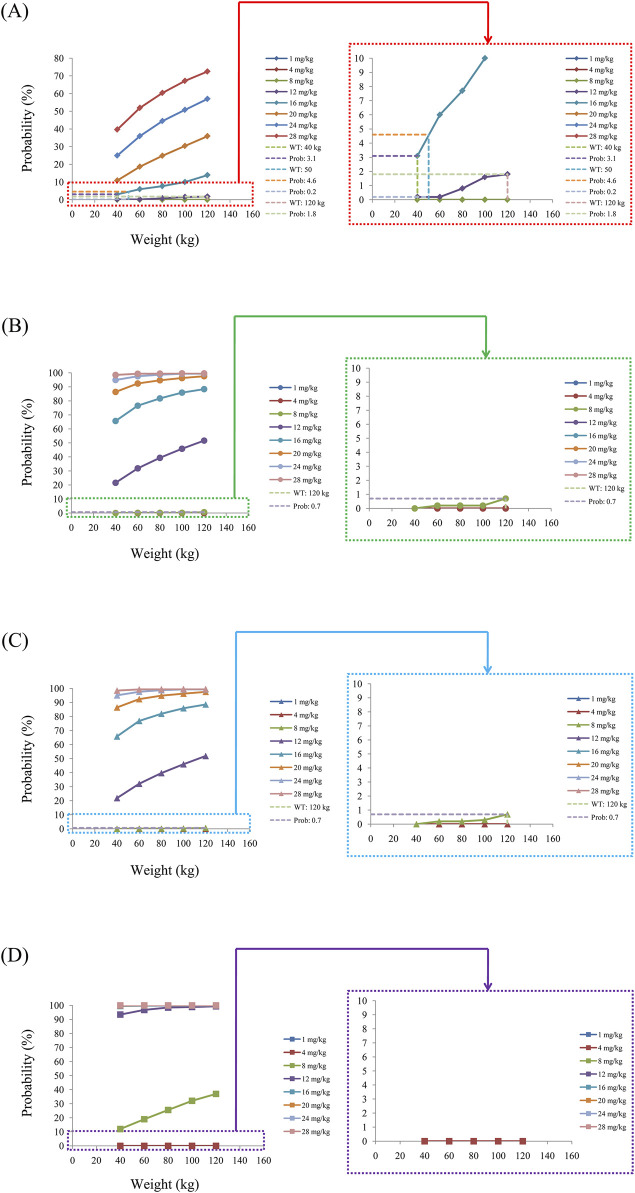
Probabilities of exceeding the upper limit of the target concentration of quetiapine in schizophrenic patients **(A)** without fluvoxamine or duloxetine coadministration, **(B)** with fluvoxamine coadministration, **(C)** with duloxetine coadministration, and **(D)** with coadministration of both fluvoxamine and duloxetine.

The probabilities of exceeding the upper limit of the treatment window (500 ng/mL) as a measure of safety under the four conditions are shown in Figure 8; here, Figures 8A–D are the schizophrenic patients without fluvoxamine or duloxetine coadministration, with fluvoxamine coadministration, with duloxetine coadministration, and with both fluvoxamine and duloxetine coadministration, respectively. For schizophrenic patients without fluvoxamine or duloxetine coadministration, the probabilities of exceeding the upper limit of the quetiapine target concentration are 3.1%–4.6% and 0.2%–1.8% when the recommended dosages are 16 and 12 mg/kg/day, respectively. For fluvoxamine coadministration, the probability of exceeding the upper limit of the quetiapine target concentration is 0%–0.7% when the recommended dosage is 8 mg/kg/day. For duloxetine coadministration, the probability of exceeding the upper limit of the quetiapine target concentration is 0%–0.7% when the recommended dosage is 8 mg/kg/day. For coadministration of both fluvoxamine and duloxetine, the probability of exceeding the upper limit of the quetiapine target concentration is 0 when the recommended dosage is 4 mg/kg/day. These data are also summarized in Table 4.

## 4 Discussion

In clinical practice, TDM is one of the important methods of guaranteeing accurate dosage of antipsychotics with low risk of adverse drug reactions and high treatment efficacy (Guo et al., 2021; Hao et al., 2023). However, the premise of this personalized drug delivery approach is that there are reference drug concentrations available for the patients from TDM; based on these known drug concentrations, the subsequent dosages of medication can be accurately adjusted to achieve the clinically needed treatment concentrations. Therefore, if there are no references from TDM for the drug concentrations administered to patients, it is not possible to recommend appropriate initial dosages for patients who are given these drugs for the first time.

PPK was used as a means to discover DDIs and achieve precise drug delivery. Here, the PPK model helped realize clinical precision of drug delivery through quantitative pharmacology, and its core intent was to promote the formulation of drug delivery protocols for clinical patients through modeling and simulation. In practical applications, Monte Carlo simulations can be combined to screen the factors influencing the course of clinical treatment, especially DDIs, and further predicting the optimal dosage based on different DDIs. The combination of PPK and Monte Carlo simulations has been widely utilized and reported for dosage recommendations (Bai et al., 2024; Deng et al., 2024; Leegwater et al., 2024; Li et al., 2024; Shen et al., 2024; Sitaruno et al., 2024; Yang et al., 2024). Therefore, we used PPK and Monte Carlo simulations in this study to analyze the clinical TDM data and patient-related information, construct a precise administration model for quetiapine in schizophrenic patients, screen the influences of DDIs, and predict the optimal initial dosage of quetiapine in schizophrenic patients based on the filtered DDI results.

In this study, we collected information from ninety-six schizophrenic patients treated with quetiapine; simultaneously, we collected the physiological and biochemical indexes of these patients along with information regarding drug combinations. By constructing the PPK model of quetiapine in schizophrenic patients, we found that the patient weight as well as fluvoxamine or duloxetine coadministration affected quetiapine clearance. The main reason for the DDIs was that quetiapine was primarily metabolized by CYP3A4 and CYP2D6 (Liu et al., 2021; Stauble et al., 2021; Rohail et al., 2023; Yau et al., 2023); however, fluvoxamine inhibited CYP3A4 (Sugahara et al., 2009; Britz et al., 2019; Huth et al., 2022) while duloxetine inhibited CYP2D6 (Ma et al., 2017; Seggio et al., 2019; Margraff et al., 2023). From the findings, we concluded that for the same weight, the quetiapine clearance rates were 1, 0.464, 0.463, and 0.214832 in schizophrenic patients without fluvoxamine or duloxetine coadministration, with fluvoxamine coadministration, with duloxetine coadministration, and with both fluvoxamine and duloxetine coadministration, respectively. Furthermore, we recommended appropriate dosages for different DDI situations. In the absence of fluvoxamine or duloxetine coadministration, 16 and 12 mg/kg/day of quetiapine are recommended to schizophrenic patients with weights in the 40–50 and 50–120 kg ranges, respectively. For fluvoxamine coadministration, 8 mg/kg/day of quetiapine is recommended to patients with weights in the 40–120 kg range. For duloxetine coadministration, 8 mg/kg/day of quetiapine is recommended to patients with weights in the 40–120 kg range. For coadministration of both fluvoxamine and duloxetine, 4 mg/kg/day of quetiapine is recommended to patients with weights in the 40–120 kg range.

Regardless of the findings, there were some limitations to this study. First, this study was a retrospective, single-center study. Second, the quetiapine concentrations were sparse sampling data from TDM. Therefore, we intend to conduct a prospective multicenter intensive sampling study in the future to further validate the recommended dosages.

## 5 Conclusion

The present study constitutes a pilot effort at investigating the potential DDIs and optimal initial dosages of quetiapine in schizophrenic patients based on PPK. Furthermore, the initial dosages of quetiapine administered to the schizophrenic patients were optimized on the basis of coadministration of fluvoxamine or duloxetine.

## Data Availability

The original contributions presented in the study are included in the article/supplementary material; further inquiries can be directed to the corresponding authors.
